# Radar Signal Sorting via a Dual-Layer Multi-Head Attention Broad Learning System

**DOI:** 10.3390/s26196201

**Published:** 2026-09-30

**Authors:** Longkun Kuang, Xuan Chen, Mingyang Du, Zhihui Li, Junpeng Shi

**Affiliations:** College of Electronic Engineering, National University of Defense Technology, Hefei 230037, China; kuanglongkun20@nudt.edu.cn (L.K.); chenxuancx@nudt.edu.cn (X.C.); lizhihui20@nudt.edu.cn (Z.L.); shijunpeng20@nudt.edu.cn (J.S.)

**Keywords:** radar signal sorting, electronic support measures, broad learning system, random orthogonal projection, incremental learning, small-sample learning

## Abstract

Radar signal sorting is a fundamental task in passive electromagnetic sensing that separates pulses emitted by multiple radars from densely interleaved streams. Existing supervised deep learning methods often require large labeled datasets and iterative optimization, limiting their applicability in non-cooperative, latency-sensitive electronic-support-measure scenarios. This article proposes a dual-layer multi-head attention broad learning system (DMHA-BLS). Conventional random feature mapping is replaced by two attention blocks with fixed random-orthogonal query, key, and value projections. A residual branch maps first-layer attention features to the input dimension through closed-form ridge regression, preserving physical pulse attributes. The output weights are also solved analytically, and accumulated Gram statistics enable batchwise updates. On an 8000-pulse simulated stream, DMHA-BLS reaches 83.17% macro F1 with 200 samples and 91.88% with 8000 samples. It provides a clear advantage over the temporal baselines in the small-sample condition, while their performance gradually approaches DMHA-BLS as the number of labeled pulses increases. In a second 1000-pulse scenario with eight emitters and strongly overlapping PDW ranges, DMHA-BLS remains the top-performing method across the evaluated sample sizes. Full-data training requires 1.57 s, compared with 100–109 s for the evaluated deep networks, indicating a favorable trade-off between sorting performance and computational efficiency.

## 1. Introduction

Radar signal sorting (RSS), also known as radar pulse deinterleaving, is a fundamental function of electronic support measures (ESM). In a typical ESM receiver, intercepted electromagnetic energy is collected and parameterized as pulse description words (PDWs) for subsequent sorting and identification. A PDW commonly contains the time of arrival (TOA), radio frequency (RF), pulse width (PW), pulse amplitude (PA), and direction of arrival (DOA) [1,2,3,4]. Modern radar sorting is challenging because emitters may use complex PRI modulations, PDW attributes from different emitters may overlap substantially, labeled pulses are limited, and ESM systems require timely processing.

Several pulse-repetition-interval (PRI) behaviors are encountered in radar emissions. Fixed PRI produces a concentrated interval pattern but can be confused by missing pulses and harmonics. Jittered PRI intentionally varies around a nominal value, which spreads the interval distribution and weakens histogram peaks. Staggered PRI cycles through several sub-PRIs, so pulse loss may destroy the periodic order even when the individual sub-PRIs are detectable. Dwell-and-switch, also called linger-and-switch, alternates between several quasi-stationary PRI states, making the switching locations difficult to determine. Sliding PRI changes continuously, causing the interval distribution to move over time rather than remain concentrated at one value. Classical TOA-based methods, including the cumulative difference histogram (CDIF), sequential difference histogram (SDIF), and PRI-transform methods, estimate candidate PRI values from interval statistics and then retrieve the corresponding pulse trains [5,6,7,8]. CDIF accumulates difference histograms over multiple orders and is interpretable, but its computational burden increases with the number of difference levels. SDIF reduces this burden by processing individual difference levels, but its reliability decreases under PRI jitter and pulse loss. PRI-transform methods can suppress harmonic ambiguities and improve the detection of jittered PRIs, but they may require higher computational cost and are less suitable for some staggered or rapidly changing PRI patterns [9,10]. Consequently, these methods are highly useful when timing regularities are prominent, but their performance may degrade when several emitters have overlapping parameters or when the intercepted stream contains substantial missing and spurious pulses.

**Figure 1:**
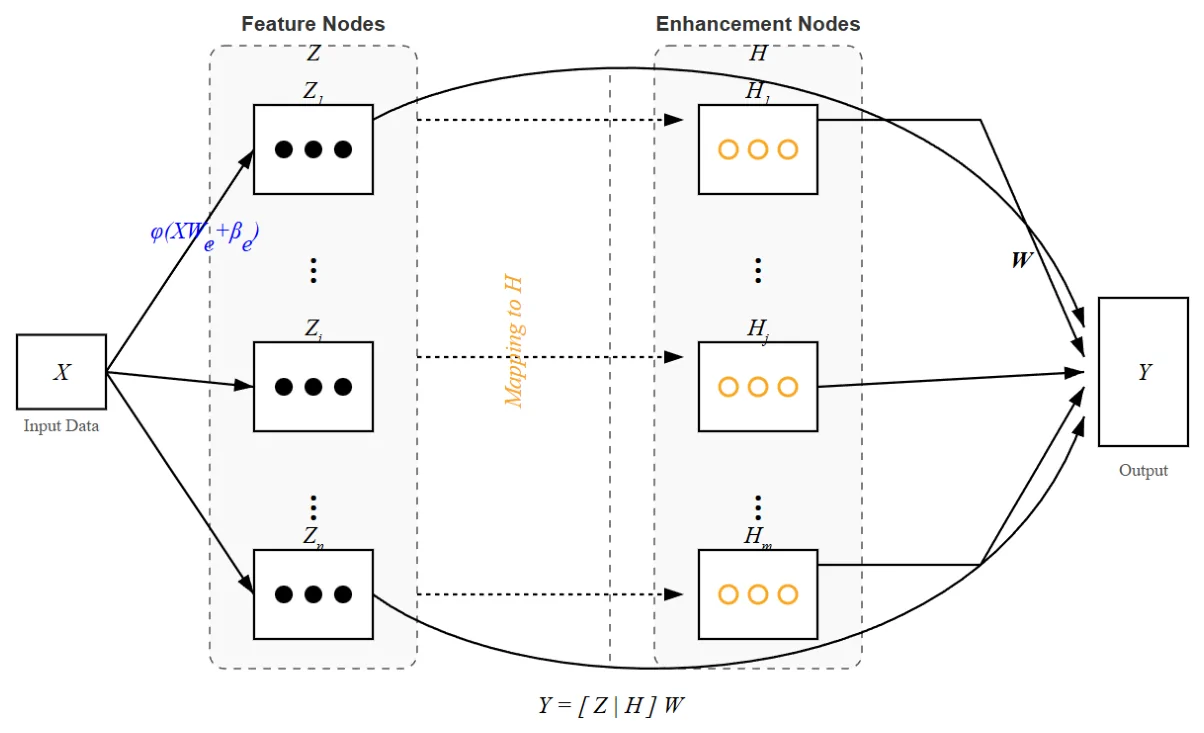
The structure of BLS.

Existing learning-based RSS methods can be viewed from three complementary perspectives. TOA- or PRI-based methods emphasize temporal regularity; multi-parameter methods use clustering or metric learning over RF, PW, DOA, and timing-related features [11,12,13,14]; and deep sequence models, including recurrent models such as bidirectional long short-term memory (BiLSTM) and bidirectional gated recurrent unit (BiGRU) and temporal convolutional models such as temporal convolutional network (TCN), learn temporal dependencies from pulse streams [15,16,17,18,19,20]. These methods provide stronger nonlinear or temporal modeling capacity, but generally require iterative optimization and a sufficiently large set of representative labeled sequences. In contrast, analytical or flat-learning models estimate output mappings in closed form. The broad learning system (BLS) (see Figure 1) is a representative example: it expands mapped and enhancement nodes without increasing network depth and supports rapid incremental updates [21]. Subsequent studies have developed fuzzy BLS, recurrent BLS, cascade-enhancement BLS, and graph-based BLS variants to improve robustness, temporal modeling, or structural representation [22,23,24,25]. Attention-BLS combinations have also been investigated for time-series prediction [26]; however, their application to radar signal sorting remains relatively limited.

In this article, we propose the Dual-Layer Multi-Head Attention Broad Learning System (DMHA-BLS) to enhance the representation capacity of baseline BLS. First, the normalized PDW vectors are processed by an MHA feature layer whose query, key, and value matrices are initialized as independent random orthogonal matrices and kept fixed thereafter. This layer computes attention within each pulse across its four PDW attributes, rather than across pulses, and extracts their interaction features while preserving the flat analytical-learning structure. Next, a residual connection with a learnable projection, solved in closed form by ridge regression, fuses the attention features with the raw PDWs to preserve the physically meaningful pulse parameters. Finally, a second MHA block with independent orthogonal projections constructs the nonlinear enhancement representation, and the output weights are solved once via the regularized pseudoinverse. The flat structure additionally admits efficient online updates upon the arrival of new pulse samples.

The principal contributions are summarized as follows:

1. A dual-layer attention BLS is introduced for small-sample radar pulse sorting. It combines attention features with a flat analytical learning architecture.
2. A random orthogonal initialization is used for the fixed query, key, and value projections. For the dimensions used in this work, these row-orthogonal mappings preserve the nonzero singular values of their input projections and reduce redundant feature directions.
3. A ridge-regression residual branch reconstructs the original PDW space between the two attention blocks, preserving physically meaningful pulse attributes without iterative training.
4. Batchwise incremental learning is derived from accumulated Gram statistics. Experiments examine small-sample accuracy, parameter sensitivity, training efficiency, pulse loss, spurious pulses, and component ablations on two simulated multi-emitter streams, including an eight-emitter scenario with strongly overlapping PDW ranges.

The remainder of this article is organized as follows. Section 2 formulates RSS and presents DMHA-BLS. Section 3 describes the experimental setup and results. Section 4 discusses the findings, practical implications, and limitations. Section 5 concludes this article.

## 2. Materials and Methods

### 2.1. Problem Formulation and Preprocessing

The objective of RSS is to correctly map each intercepted pulse to its originating emitter identity. Consider *C* active radar emitters whose pulses are interleaved in a single intercepted stream. Pulse *i* is represented by

(1) $$p_{i}={\left[{\mathrm{DTOA}}_{i},\;{\mathrm{RF}}_{i},\;{\mathrm{PW}}_{i},\;{\mathrm{DOA}}_{i}\right]}^{⊤}\in R^{4},$$

where DTOA is a local inter-arrival descriptor computed from the chronological interleaved stream; in a multi-emitter stream, it is not generally the true emitter-specific PRI. Moreover, to reduce input variance and improve stationarity without discarding pulse-stream information, DTOA is often used as a proxy for TOA in radar-sorting algorithms; this substitution can improve computational efficiency and input stability [20]. PA is excluded because its value can vary strongly with propagation, receiver gain, and aspect. Given the labeled set

(2) $$D={\left\{\left(p_{i},y_{i}\right)\right\}}_{i=1}^{N},$$

RSS is treated as a *C*-class classification problem. The input matrix is $P={\left[p_{1},\ldots ,p_{N}\right]}^{⊤}\in R^{N\times D}$, with D=4, and the desired mapping is

(3) $$\hat{Y}=F\left(P;\Theta \right),$$

where Θ denotes the model parameters and $\hat{Y}$ contains the predicted class scores. A key challenge addressed in this work is that the training set size $N_{\mathrm{train}}$ is assumed to be small, requiring the model F to possess high generalization capability and rapid convergence. DTOA is computed from adjacent pulses after ordering the chronological interleaved reception stream and before emitter labels are used; it is not constructed from per-emitter pulse sequences.

DTOA, RF, PW, and DOA have different physical scales (e.g., GHz for RF vs. μs for PW). To ensure numerical stability and balanced contribution from all features, we apply min–max normalization to standardize the input. Each dimension *j* of the feature vector is scaled to the range [0,1]:

(4) $$x_{i,j}=\frac{p_{i,j}-p_{j}^{\min}}{p_{j}^{\max}-p_{j}^{\min}+\epsilon },$$

where $p_{j}^{\min}$ and $p_{j}^{\max}$ are estimated from the training subset and ϵ prevents division by zero. The same statistics are then applied to validation or test data. The normalized matrix is denoted by $X\in R^{N\times D}$.

### 2.2. Architecture Overview

**Figure 2:**
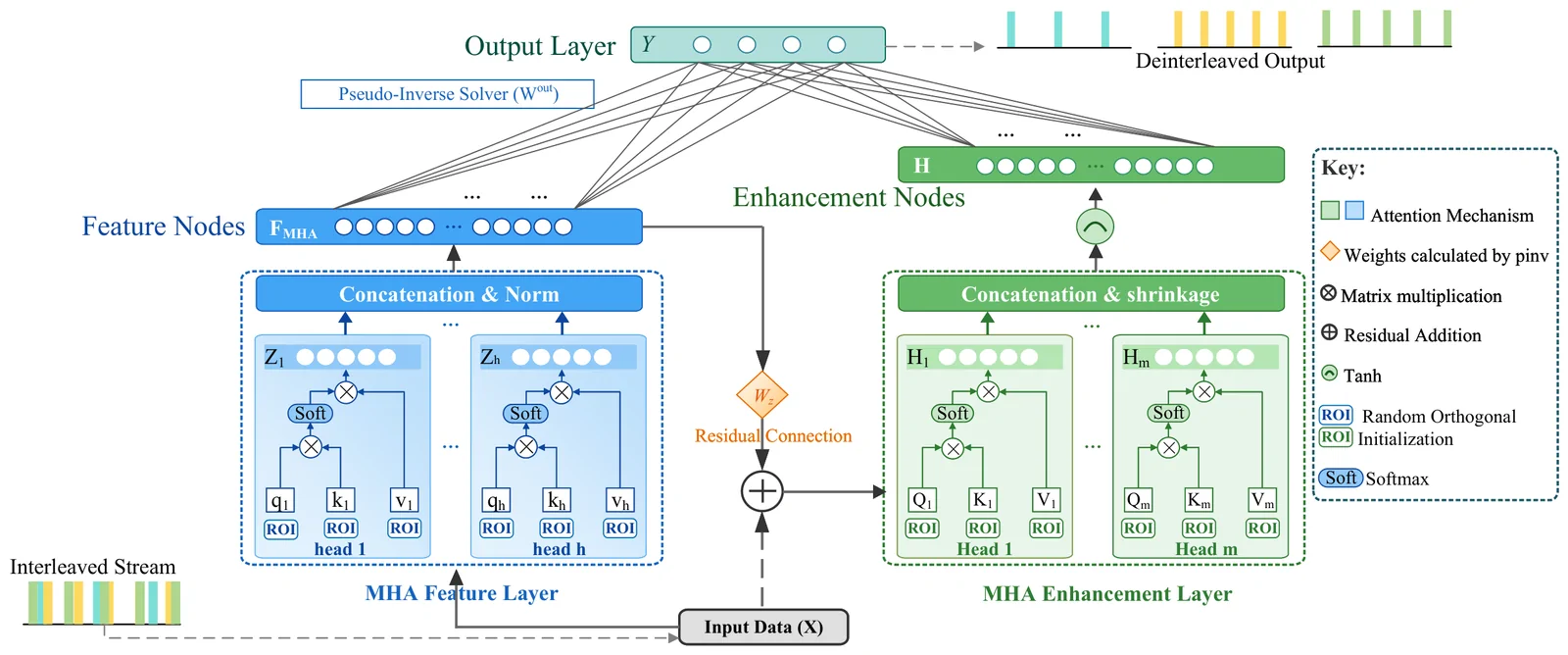
Architecture of DMHA-BLS. Two fixed random-orthogonal MHA blocks are connected through a closed-form residual projection. The final output weights are obtained analytically by ridge regression.

RSS is formulated as supervised, pulse-wise classification: *C* is fixed and known, labeled pulses are available, and each target pulse receives one emitter label. The per-pulse data flow is $D=4\to S_{i}\in R^{4\times 12}\to 144\to 4\to 144\to p=288\to C$. Figure 2 shows the proposed architecture. First, normalized PDW vectors are processed by an MHA feature layer whose query, key, and value matrices are initialized as independent random orthogonal matrices and then fixed. Next, a residual branch maps the first-layer attention features back to the input dimension by closed-form ridge regression and fuses them with the raw normalized PDWs. A second MHA block with an independent set of orthogonal projections constructs a nonlinear enhancement representation. Finally, the feature and enhancement nodes are concatenated, and the output weights are solved once through regularized least squares. Thus, DMHA-BLS retains the flat, non-iterative character of BLS while introducing attention feature extraction across the four PDW attributes.

### 2.3. Random Orthogonal Projection Initialization

Standard BLS and random-vector functional-link models commonly draw input projections independently from a Gaussian distribution. The spread of the resulting singular values can produce redundant directions and poorly conditioned regression matrices. DMHA-BLS instead initializes every attention projection through a QR factorization.

For a target matrix $W\in R^{d_{\mathrm{in}}\times d_{\mathrm{out}}}$, when $d_{\mathrm{in}}\geq d_{\mathrm{out}}$, a Gaussian matrix $G\in R^{d_{\mathrm{in}}\times d_{\mathrm{out}}}$ is sampled and factorized as G=QR. Setting W=Q gives $W^{⊤}W=I_{d_{\mathrm{out}}}$. When $d_{\mathrm{in}}<d_{\mathrm{out}}$, the same operation is applied to the transposed shape and $W=Q^{⊤}$, yielding

(5) $$WW^{⊤}=I_{d_{\mathrm{in}}}.$$

For any X and row-orthogonal W, $\left(XW\right){\left(XW\right)}^{⊤}=XX^{⊤}$. Hence, if $\sigma_{i}^{+}\left(\cdot \right)$ denotes a nonzero singular value, then $\sigma_{i}^{+}\left(XW\right)=\sigma_{i}^{+}\left(X\right)$, and the effective spectral condition number satisfies

$$\kappa_{2}^{+}\left(XW\right)=\frac{\sigma_{\max}\left(XW\right)}{\sigma_{\min}^{+}\left(XW\right)}=\kappa_{2}^{+}\left(X\right).$$

Moreover, each square-orthogonal query, key, or value projection has singular values all equal to one and therefore $\kappa_{2}\left(W\right)=1$. By contrast, an unconstrained Gaussian projection has realization-dependent singular values and provides no identity of the form $WW^{⊤}=I$; it may therefore expand or contract different directions. This is a stability guarantee for the linear projections, not a claim that every Gaussian realization must yield inferior classification. In addition, the attention pattern of head *r* depends on the bilinear operator $W_{r}^{Q}W_{r}^{K⊤}$. Independent draws across heads produce distinct mixing operators, while the orthogonality constraint limits degeneracy within each projection. As all projections are fixed and never trained, this well-conditioned diversity is important in the small-sample regime. The controlled comparison between Gaussian and orthogonal projections is reported in Section 3.8.

### 2.4. First Multi-Head Attribute-Attention Feature Layer

The first attention block applies sample-wise self-attention across the PDW attributes of each pulse. From an electromagnetic-signal perspective, RF, PW, DOA, and TOA/DTOA are multi-domain projections of the same pulse across the transmission, propagation, and reception chain [1,2]. The array manifold depends on frequency, PW affects detectability and effective SNR, receiver group delay can vary with frequency, and DOA and TOA/DTOA are coupled through propagation geometry and motion. Their conditional joint distribution is therefore generally non-factorized. After training-only min–max normalization and shared embedding, self-attention can learn pairwise and higher-order non-diagonal interactions among timing, frequency, pulse-width, and bearing signatures. In this sense, cross-attribute attention represents measurement coupling, waveform-mode constraints, and temporal-geometric constraints that independent feature weighting may discard. Its attention weights represent interaction features among the current pulse’s DTOA, RF, PW, and DOA. For pulse *i*, let

(6) $$x_{i}={\left[x_{i,1},\ldots ,x_{i,D}\right]}^{⊤}\in R^{D}$$

denote its normalized PDW vector. A fixed random embedding matrix $E\in R^{D\times d_{k}}$ converts the *D* scalar attributes into *D* attribute tokens:

(7) $$S_{i}=\mathrm{diag}\left(x_{i}\right)E,\;S_{i}\in R^{D\times d_{k}}.$$

The *a*-th row of $S_{i}$ is the embedded representation of the *a*-th PDW attribute of pulse *i*.

For attention head r=1,…,h, fixed square-orthogonal matrices $W_{r}^{Q}$, $W_{r}^{K}$, and $W_{r}^{V}\in R^{d_{k}\times d_{k}}$ generate

(8) $$Q_{i,r}=S_{i}W_{r}^{Q},\;K_{i,r}=S_{i}W_{r}^{K},\;V_{i,r}=S_{i}W_{r}^{V}.$$

**Figure 3:**
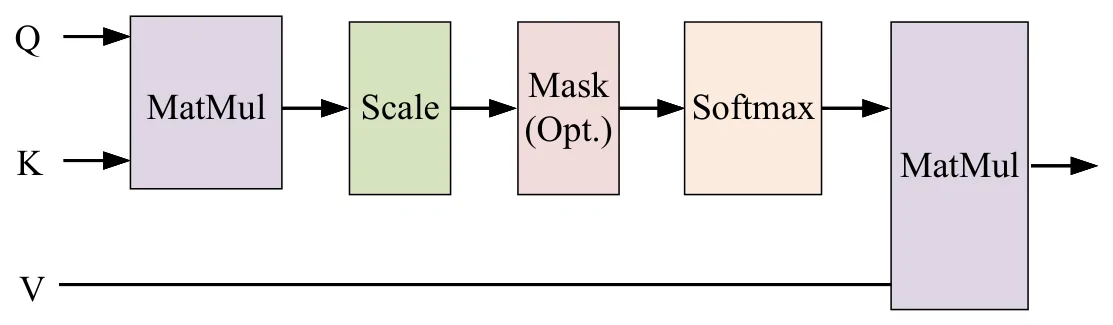
The architecture of self-attention.

Scaled dot-product attention, as shown in Figure 3, is then calculated across the *D* attribute tokens:

(9) $$U_{i,r}=\mathrm{softmax}\left(\frac{Q_{i,r}K_{i,r}^{⊤}}{\sqrt{d_{k}}}\right)V_{i,r},\;U_{i,r}\in R^{D\times d_{k}},$$

where the softmax is applied row-wise. Accordingly, the attention matrix has dimension D×D; with the four PDW attributes used in this study, its dimension is 4×4.

The output of each head is reduced to a $d_{k}$-dimensional vector through coordinate-wise maximum pooling over the attribute-token dimension:

(10) $$z_{i,r,b}=\max_{a\in \{1,\ldots ,D\}}U_{i,r,a,b},\;b=1,\ldots ,d_{k}.$$

Let $z_{i,r}={\left[z_{i,r,1},\ldots ,z_{i,r,d_{k}}\right]}^{⊤}$. Collecting the outputs of head *r* for all pulses gives

(11) $$Z_{r}=\left[\begin{array}{c} z_{1,r}^{⊤}\\ \vdots \\ z_{N,r}^{⊤} \end{array}\right]\in R^{N\times d_{k}}.$$

The coordinates of each head are normalized independently using statistics estimated only from the training set. For head *r* and coordinate *b*, define

(12) $$\ell_{r,b}=\min_{i\in D_{\mathrm{train}}}z_{i,r,b},\;u_{r,b}=\max_{i\in D_{\mathrm{train}}}z_{i,r,b},$$

and

(13) $$\delta_{r,b}=\left\{\begin{array}{cc} u_{r,b}-\ell_{r,b},\; & u_{r,b}>\ell_{r,b},\\ 1,\; & u_{r,b}=\ell_{r,b}. \end{array}\right.$$

The normalized feature is

(14) $${\hat{z}}_{i,r,b}=\frac{z_{i,r,b}-\ell_{r,b}}{\delta_{r,b}}.$$

The training-set values of $\ell_{r,b}$ and $\delta_{r,b}$ are stored and reused for validation and test pulses.

Finally, the normalized head outputs are concatenated:

(15) $$F_{\mathrm{MHA}}=\left[{\hat{Z}}_{1},\ldots ,{\hat{Z}}_{h}\right]\in R^{N\times hd_{k}}.$$

Each row of $F_{\mathrm{MHA}}$ depends only on the corresponding pulse.

### 2.5. Closed-Form Residual Projection

Although the first MHA layer captures interactions among the PDW attributes of each pulse, repeated feature transformations may obscure physically meaningful PDW attributes, such as the precise DTOA, RF, PW, and DOA values required for emitter discrimination. A conventional residual connection cannot be directly applied because the concatenated attention representation $F_{\mathrm{MHA}}\in R^{N\times hd_{k}}$ and the normalized input $X\in R^{N\times D}$ have different feature dimensions. We therefore introduce a learnable linear projection to align the two feature spaces before residual fusion.

The core idea is to learn a linear mapping that projects the abstract MHA features back into the original input space, effectively reconstructing an approximation of the raw signal from its deep representation. Let $W_{\mathrm{res}}\in R^{hd_{k}\times D}$ denote the reconstruction matrix. It is estimated by solving the ridge-regression problem

(16) $$\underset{W}{\mathrm{minimize}}\;{∥F_{\mathrm{MHA}}W-X∥}_{F}^{2}+\lambda_{\mathrm{res}}{∥W∥}_{F}^{2},$$

where $\lambda_{\mathrm{res}}$ controls the strength of the $L_{2}$ regularization. Setting the gradient to zero gives the normal equation

$$\left(F_{\mathrm{MHA}}^{⊤}F_{\mathrm{MHA}}+\lambda_{\mathrm{res}}I\right)W_{\mathrm{res}}=F_{\mathrm{MHA}}^{⊤}X,$$

and therefore the closed-form solution

(17) $$W_{\mathrm{res}}={\left(F_{\mathrm{MHA}}^{⊤}F_{\mathrm{MHA}}+\lambda_{\mathrm{res}}I\right)}^{-1}F_{\mathrm{MHA}}^{⊤}X.$$

This formulation is ordinary multi-output ridge regression rather than an inverse model in the signal-processing sense. The target is deliberately X because the projected branch must return to the *D*-dimensional physical PDW coordinate space before it can be added componentwise to the raw-input branch. A direct concatenation $\left[F_{\mathrm{MHA}},X\right]$ would preserve the input but would leave heterogeneous, unaligned coordinates and increase the feature width supplied to the second attention block. A conventional trainable ResNet projection would require iterative backpropagation and introduce additional data-dependent parameters. In contrast, ridge regression preserves the analytical-learning design and gives a unique solution for every $\lambda_{\mathrm{res}}>0$. In particular, the coefficient matrix is positive definite, with

$$\kappa_{2}\;\left(F_{\mathrm{MHA}}^{⊤}F_{\mathrm{MHA}}+\lambda_{\mathrm{res}}I\right)=\frac{\sigma_{\max}^{2}\left(F_{\mathrm{MHA}}\right)+\lambda_{\mathrm{res}}}{\sigma_{\min}^{2}\left(F_{\mathrm{MHA}}\right)+\lambda_{\mathrm{res}}},$$

where $\sigma_{\min}$ includes a possible zero singular value. Thus, the smallest eigenvalue is at least $\lambda_{\mathrm{res}}$, preventing singular normal equations. Equation (17) states the analytical solution compactly; in implementation, the corresponding linear system is solved directly rather than by explicitly forming a matrix inverse.

The projected attention features are subsequently fused with the original input:

(18) $$Z_{\mathrm{res}}=F_{\mathrm{MHA}}W_{\mathrm{res}}+X,\;Z_{\mathrm{res}}\in R^{N\times D}.$$

The residual structure serves two complementary purposes. Structurally, it bridges the dimensional mismatch between the broad attention representation and the original PDW space. Functionally, the direct branch preserves the raw pulse attributes, while the projected branch introduces information derived from within-pulse attribute interactions. Consequently, $Z_{\mathrm{res}}$ remains physically grounded while incorporating nonlinear relationships among DTOA, RF, PW, and DOA, providing a more informative input to the subsequent enhancement layer.

### 2.6. Enhancement Layer and Analytical Output Weights

The residual representation $Z_{\mathrm{res}}$ is passed to a second MHA block in the same per-pulse manner as the first MHA layer, yielding nonlinear enhancement features. Its query, key, and value matrices are drawn independently of those in the first block using the random orthogonal initialization described in Section 2.3. The independent projections encourage the two blocks to capture complementary pulse relationships.

For the *k*-th enhancement head, k=1,…,m, the operation is denoted by ${\mathrm{Attn}}_{k}\left(Z_{\mathrm{res}}\right)$. The outputs of the *m* heads are concatenated as

(19) $$O_{\mathrm{enh}}=\left[{\mathrm{Attn}}_{1}\left(Z_{\mathrm{res}}\right),\ldots ,{\mathrm{Attn}}_{m}\left(Z_{\mathrm{res}}\right)\right]\in R^{N\times md_{k}}.$$

A bounded nonlinear transformation is then applied to construct the enhancement nodes:

(20) $$H=\tanh\left(sO_{\mathrm{enh}}+\beta \right),\;H\in R^{N\times md_{k}},$$

where *s* is the scaling factor and $\beta \in R^{1\times md_{k}}$ is a bias vector broadcast across all pulse samples. In this study, the bias entries are independently initialized from U(−1,1) and remain fixed. The scaling factor controls the scale of the attention features before activation, while the hyperbolic tangent function introduces a zero-centered and bounded nonlinearity. This transformation broadens the representation space and helps describe nonlinear decision boundaries between overlapping emitter classes.

The first-layer attention features and nonlinear enhancement features are horizontally concatenated to form the broad system matrix

(21) $$A=\left[F_{\mathrm{MHA}}|H\right]\in R^{N\times p},\;p=\left(h+m\right)d_{k}.$$

Thus, the final classifier simultaneously receives the attention features generated from the original PDWs and the nonlinear features generated from the residual representation.

Given the one-hot label matrix $Y\in R^{N\times C}$, the output matrix $W^{\mathrm{out}}\in R^{p\times C}$ is estimated through the regularized least-squares objective

(22) $$\underset{W^{\mathrm{out}}}{\mathrm{minimize}}\;{∥AW^{\mathrm{out}}-Y∥}_{F}^{2}+\lambda {∥W^{\mathrm{out}}∥}_{F}^{2},$$

where λ controls the trade-off between fitting the training labels and limiting the magnitude of the output weights. The convex objective in Equation (22) has the analytical solution

(23) $$W^{\mathrm{out}}={\left(A^{⊤}A+\lambda I\right)}^{-1}A^{⊤}Y.$$

For an input pulse batch, the class-score matrix is calculated as

(24) $$\hat{Y}=AW^{\mathrm{out}},$$

and the predicted emitter of pulse *i* is

(25) $${\hat{y}}_{i}=\underset{c\in \{1,\ldots ,C\}}{\arg\max}\;{\hat{Y}}_{i,c}.$$

Because all attention projections and biases remain fixed, DMHA-BLS training requires only the two convex ridge-regression solves in Equations (17) and (23). This avoids epoch-wise backpropagation and local-minimum-related optimization behavior. In practice, both systems are evaluated using a numerically stable linear solver rather than explicitly forming the matrix inverse. The resulting architecture provides rapid model fitting and updating relative to iterative deep networks.

### 2.7. Training and Inference Procedure

For clarity, the complete offline learning procedure is summarized below:

1. Normalize the PDW dimensions using Equation (4) and store the training-set scaling parameters.
2. Generate the first-layer query, key, and value matrices using the orthogonal initialization in Section 2.3; keep them fixed and compute $F_{\mathrm{MHA}}$.
3. Estimate $W_{\mathrm{res}}$ using Equation (17) and form $Z_{\mathrm{res}}$.
4. Independently initialize the second MHA block, compute $O_{\mathrm{enh}}$, and apply Equation (20) to obtain H.
5. Concatenate $F_{\mathrm{MHA}}$ and H, then solve Equation (23) for $W^{\mathrm{out}}$.
6. At inference, reuse the training-set scaling parameters and all fixed projections, apply the learned residual projection, construct the broad features, and assign each pulse to the class with the largest output score.

No gradient, epoch schedule, or iterative parameter update is required. The only data-dependent matrix estimates are $W_{\mathrm{res}}$ and $W^{\mathrm{out}}$.

### 2.8. Incremental Update

A pivotal advantage of the proposed DMHA-BLS framework over traditional deep learning models is its inherent capability for incremental learning. This allows the system to update its decision boundaries batchwise when new labeled data arrive, without retraining the entire network from scratch. Define the sufficient statistics

(26) $$G=A^{⊤}A,\;B=A^{⊤}Y.$$

When a new labeled batch $\left(X_{\mathrm{new}},Y_{\mathrm{new}}\right)$ arrives, the fixed attention projections and frozen $W_{\mathrm{res}}$ generate $A_{\mathrm{new}}$. The min–max and head-normalization statistics, attention projections, biases, and $W_{\mathrm{res}}$ all retain their initial-training values; only G, B, and consequently $W^{\mathrm{out}}$ are updated. The statistics are updated by

(27) $$\begin{array}{cc} G & \leftarrow G+A_{\mathrm{new}}^{⊤}A_{\mathrm{new}},\\ B & \leftarrow B+A_{\mathrm{new}}^{⊤}Y_{\mathrm{new}}, \end{array}$$

and

(28) $$W_{\mathrm{new}}^{\mathrm{out}}={\left(G+\lambda I\right)}^{-1}B.$$

The update requires $O(N_{\mathrm{new}}p^{2}+p^{3})$ operations and $O(p^{2})$ persistent memory. The broad feature vector of each pulse is independent of the other pulses in the incoming batch after DTOA construction; at a batch boundary, DTOA is computed using the immediately preceding pulse rather than resetting the sequence. Consequently, Equations (27) and (28) recover the exact ridge-regression output solution for the accumulated broad features generated by this frozen mapping. This equivalence is conditional on the frozen feature mapping and does not imply equivalence to an offline model that re-estimates the scaling statistics or residual projection using all accumulated samples. Thus, the update is classifier adaptation in a fixed feature space, rather than end-to-end continual representation learning. It assumes labeled incoming batches, unchanged PDW definitions and known emitter classes, and no severe distribution or sensor-calibration shift that invalidates the frozen scaling and residual mapping; such drift or novel emitters would require re-estimation beyond the present update rule.

### 2.9. Evaluation Metrics

Radar signal sorting is a multi-class classification problem in which the numbers of intercepted pulses associated with different emitters may be unequal. Therefore, class-wise precision, recall, and F1-score are first calculated independently for each emitter, thereby preventing emitters with more pulses from dominating the overall evaluation.

For emitter class *c*, the precision and recall are defined as

(29) $$P_{c}=\frac{TP_{c}}{TP_{c}+FP_{c}},\;R_{c}=\frac{TP_{c}}{TP_{c}+FN_{c}},$$

where $TP_{c}$, $FP_{c}$, and $FN_{c}$ denote the corresponding classification counts for emitter *c*. The class-wise F1-score is the harmonic mean of precision and recall:

(30) $$F1_{c}=\frac{2P_{c}R_{c}}{P_{c}+R_{c}}.$$

Given *C* radar emitter classes, the macro-averaged F1-score is calculated as

(31) $$F1_{\mathrm{macro}}=\frac{1}{C}\sum_{c=1}^{C}F1_{c}.$$

It should be noted that $F1_{\mathrm{macro}}$ is obtained by averaging the individual class-wise F1-scores rather than by directly taking the harmonic mean of $P_{\mathrm{macro}}$ and $R_{\mathrm{macro}}$. Consequently, every emitter contributes equally to the final score, regardless of the number of pulses assigned to that class. The macro F1-score is used as the principal evaluation metric in the following experiments.

## 3. Results

### 3.1. Simulation Dataset

**Figure 4:**
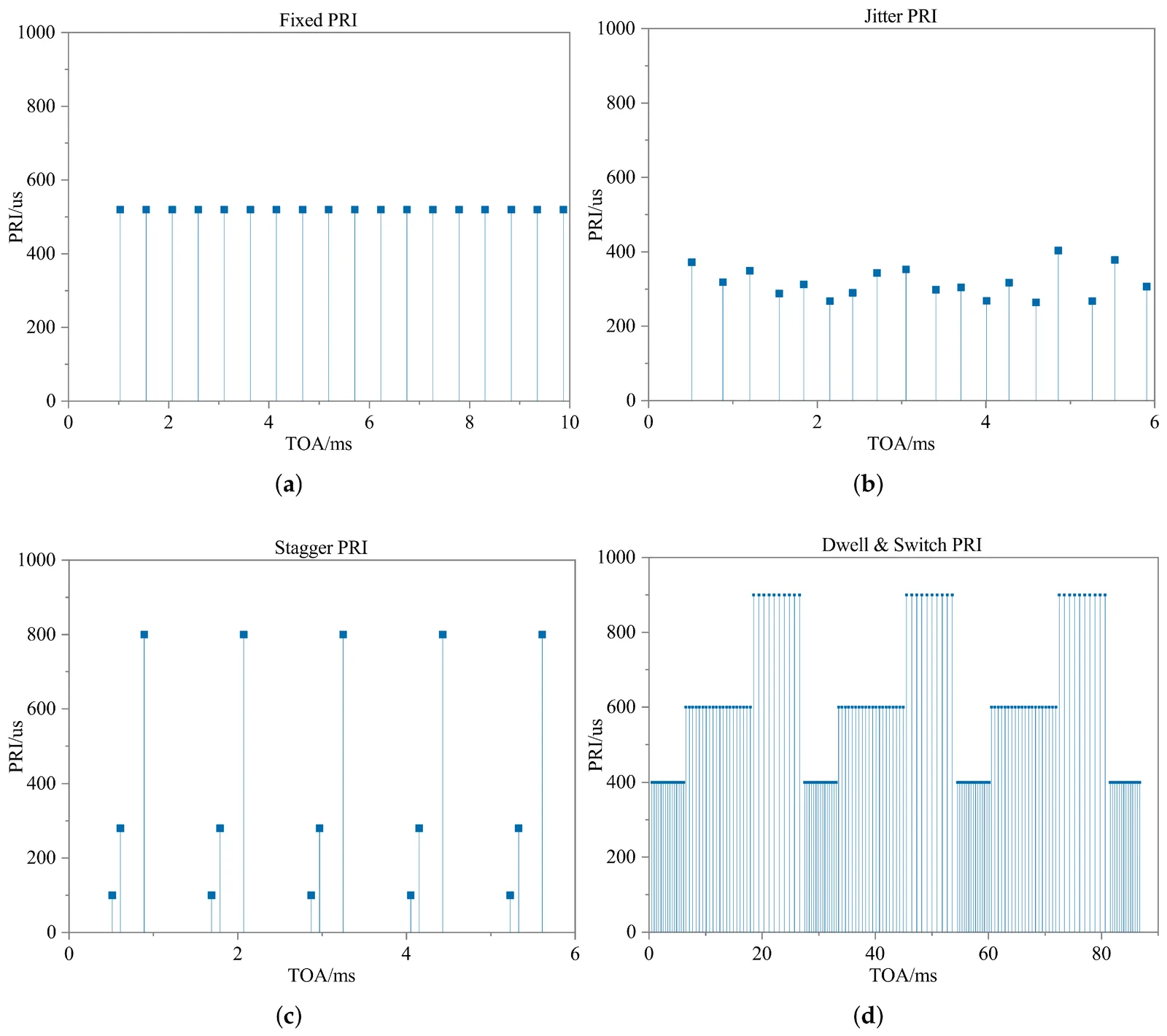
Four typical PRI modulations. (**a**) Fixed PRI. (**b**) Jitter PRI. (**c**) Stagger PRI. (**d**) Dwell & Switch PRI.

Radar PDW parameters are typically proprietary, and public measured datasets that provide pulse-level PDWs, emitter labels, and acquisition order are scarce in the RSS domain. We therefore use reproducible simulated streams. The primary four-emitter parameter setting and 4:1 data partition follow the simulation protocol in [20]. Specifically, the dataset contains four representative emitters employing distinct PRI modulation schemes to mimic modern radar agility, namely Fixed, Jittered, Staggered, and Dwell & Switch, whose schematic patterns are illustrated in Figure 4. The corresponding PRI configurations are summarized in Table 1: Fixed PRI is set to 520 μs; Jitter PRI adopts a base PRI of 350 μs with a jitter coefficient J=0.25; Staggered PRI follows the PRI sequence {100,280,800} μs; and Dwell & Switch PRI uses the pattern 400 μs × 15, 600 μs × 20, and 900 μs × 10. The RF values all employ jittered modulation. For each emitter, additional PDW attributes such as PW and DOA are randomly sampled within predefined ranges to diversify intra-class variations.

The resulting stream spanned approximately 1 s and contained 8000 pulses, corresponding to an average intercepted-stream density of about 8000 pulses/s. Unless otherwise specified, every sample-size experiment used a strict chronological 4:1 split: the first 80% of the selected stream formed the training subset and the final 20% formed the test subset, without random shuffling across the split boundary. The reported number of samples denotes the total number before this split. All normalization statistics and data-dependent model parameters were estimated from the training subset only. All methods were evaluated on the same eligible test-target indices. The pulse-loss and spurious-pulse experiments serve as controlled proxies for missed detections and false alarms. This protocol was applied to both the four-emitter stream and the additional eight-emitter stream described in Section 3.3.

### 3.2. Experimental Configuration

#### 3.2.1. Compared Methods and Evaluation Protocol

The proposed DMHA-BLS was compared with eight representative methods: cumulative difference histogram (CDIF) [5], *K*-nearest neighbors (KNN) [13], temporal convolutional network (TCN) [20], bidirectional long short-term memory network (BiLSTM) [18], bidirectional gated recurrent unit network (BiGRU) [17], standard BLS [21], and constrained-seed K-means (CS-K) [27]. CDIF represents a classical TOA-based baseline, KNN provides a non-parametric classifier on the same four-dimensional PDW input, and the other methods represent temporal convolution, recurrent sequence modeling, analytical broad learning, and conventional clustering, respectively. CDIF estimates candidate PRI intervals from the interleaved TOA stream and retrieves the corresponding pulse trains; its pulse-level outputs are aligned with the ground-truth emitter labels for macro-F1 evaluation. Its implementation used a 1μs histogram bin, a 40–1500μs PRI search range, threshold coefficient k=0.5, a maximum accumulated difference order of 50, and a sequence confirmation requirement of at least five consecutive matched pulses. KNN used K=C, Euclidean distance, and uniform neighbor weights. For CS-K, the resulting cluster indices were aligned with the ground-truth emitter labels using the Hungarian algorithm.

All methods received the same normalized PDW attributes, namely DTOA, RF, PW, and DOA, and produced one emitter label for each target pulse. DMHA-BLS, BLS, KNN, and CS-K operated on the current pulse. For KNN, the training pulses were retained as the reference set and the test labels were assigned by the K=C nearest training vectors. To evaluate the temporal models under an architecture-appropriate input configuration, TCN, BiLSTM, and BiGRU received a trailing window of 16 chronologically ordered PDWs comprising the target pulse and its preceding 15 pulses. These windows were constructed separately inside the training and test subsets after the chronological split; any window crossing the split boundary was discarded. Consequently, no training pulse occurs in a test window, no target is predicted using a future pulse, and the same eligible target indices are used for all methods. The corresponding label was that of the final pulse in the window. For TCN, the input tensor was arranged as (B,4,16), whereas BiLSTM and BiGRU used (B,16,4). The normalization parameters were estimated from the training subset and subsequently applied to the test subset. The deep networks were trained using the cross-entropy loss and Adam optimizer for 200 epochs with a batch size of 32.

All methods were evaluated over 10 independent runs with independently varied random seeds, and the results are reported as the mean and standard deviation. For DMHA-BLS, each run regenerates the fixed random-orthogonal projections and recomputes the closed-form solutions; the repeated-run statistics therefore include random-initialization variability as well as training stochasticity. Experiments used Windows 11 Pro 25H2 on an Intel Core i9-14900KF workstation with 128 GB RAM and an NVIDIA RTX 4090 GPU with 24 GB memory. The software environment was Python 3.9.21, PyTorch 2.6.0+cu126, NumPy 2.0.2, SciPy 1.13.1, and scikit-learn 1.6.1; the temporal networks ran on the GPU, whereas DMHA-BLS, BLS, and CS-K ran on the CPU. Wall-clock times were averaged over the same 10 runs. Training was timed from model initialization, after the normalized training tensors were available, through completion of all 200 epochs or the analytical solution; testing was timed from the prepared test tensors through production of all class scores. Data generation, disk input/output, splitting, normalization, and metric calculation were excluded.

#### 3.2.2. DMHA-BLS and BLS Configuration

The principal settings of DMHA-BLS and standard BLS are summarized in Table 2. DMHA-BLS used 12 heads in both the feature and enhancement layers, with a per-head dimension of 12. The two attention blocks therefore generated 144 feature nodes and 144 enhancement nodes, resulting in a concatenated broad width of 288. Standard BLS used 24 feature windows with 36 nodes per window and 800 enhancement nodes.

#### 3.2.3. Deep Learning Baseline Configuration

The implemented TCN contained two temporal residual blocks with channel widths of 32 and 64. Each block comprised two dilated causal convolutional layers, ReLU activations, dropout, and a residual connection. Dilation factors of 1 and 2 were used in the two blocks, respectively. The representation at the final temporal position was passed to the *C*-class classifier so that the output corresponded to the last pulse of the trailing window, where *C* is the number of emitters in the evaluated scenario.

BiLSTM and BiGRU both contained two stacked bidirectional recurrent layers with 64 hidden units in each direction. The resulting 128-dimensional bidirectional representation at the final sequence position was processed by dropout and a fully connected classifier. BiLSTM employed separate memory and hidden states, whereas BiGRU used reset and update gates, resulting in fewer trainable parameters. Their detailed configurations are compared with the TCN settings in Table 3.

#### 3.2.4. Complexity and Model Size

To complement the wall-clock measurements, Table 4 reports architecture-level parameter counts and asymptotic costs. The counts use the final reported four-emitter configuration (C=4), temporal-window length L=16, four PDW inputs per time step, DMHA-BLS widths $q=hd_{k}=144$ and p=288, and standard bias terms in the temporal baselines. Here, *N* denotes the number of target windows, E=200 the number of training epochs, and $P_{m}$ the per-time-step arithmetic cost of baseline *m* determined by its implemented convolutional or recurrent layers. Fixed random projections are included in stored model size, whereas the residual and output ridge solutions are listed as solved parameters. Optimizer states and transient batch buffers are excluded.

For DMHA-BLS, the persistent incremental-update state is $O(p^{2}+pC)$, in addition to the fixed projections and normalization statistics. The approximately $5.1\times 10^{4}$ per-pulse operations include two 12-head attention blocks with D=4 attribute tokens, the residual projection, and the final class-score multiplication. These analytical counts describe the implemented architecture and are complementary to the measured runtime comparison.

#### 3.2.5. KNN Baseline Configuration

KNN was included as a direct non-parametric reference on the identical normalized four-dimensional pulse-wise input. The number of neighbors was fixed to K=C, where *C* is the known number of emitters in the scenario; Euclidean distance and uniform neighbor weights were used. The chronological training/test split and training-only normalization were unchanged. KNN stores the training vectors and labels rather than estimating an iterative model, so its inference cost grows linearly with the number of retained training pulses.

#### 3.2.6. Conventional Clustering Baseline Configuration

CS-K was included as a conventional clustering baseline. For each independent run, 10% of the training samples from each emitter were randomly selected as labeled seeds, and their class-wise mean vectors were used to initialize the cluster centers. K-means was then performed using *C* clusters, where *C* is the number of emitters in the scenario, with one centroid initialization and a maximum of 300 iterations. Cluster indices were aligned with emitter labels using the Hungarian algorithm on the training subset, and the resulting mapping was kept fixed during test-set evaluation. The detailed settings are listed in Table 5.

### 3.3. Small-Sample Sorting Performance

**Figure 5:**
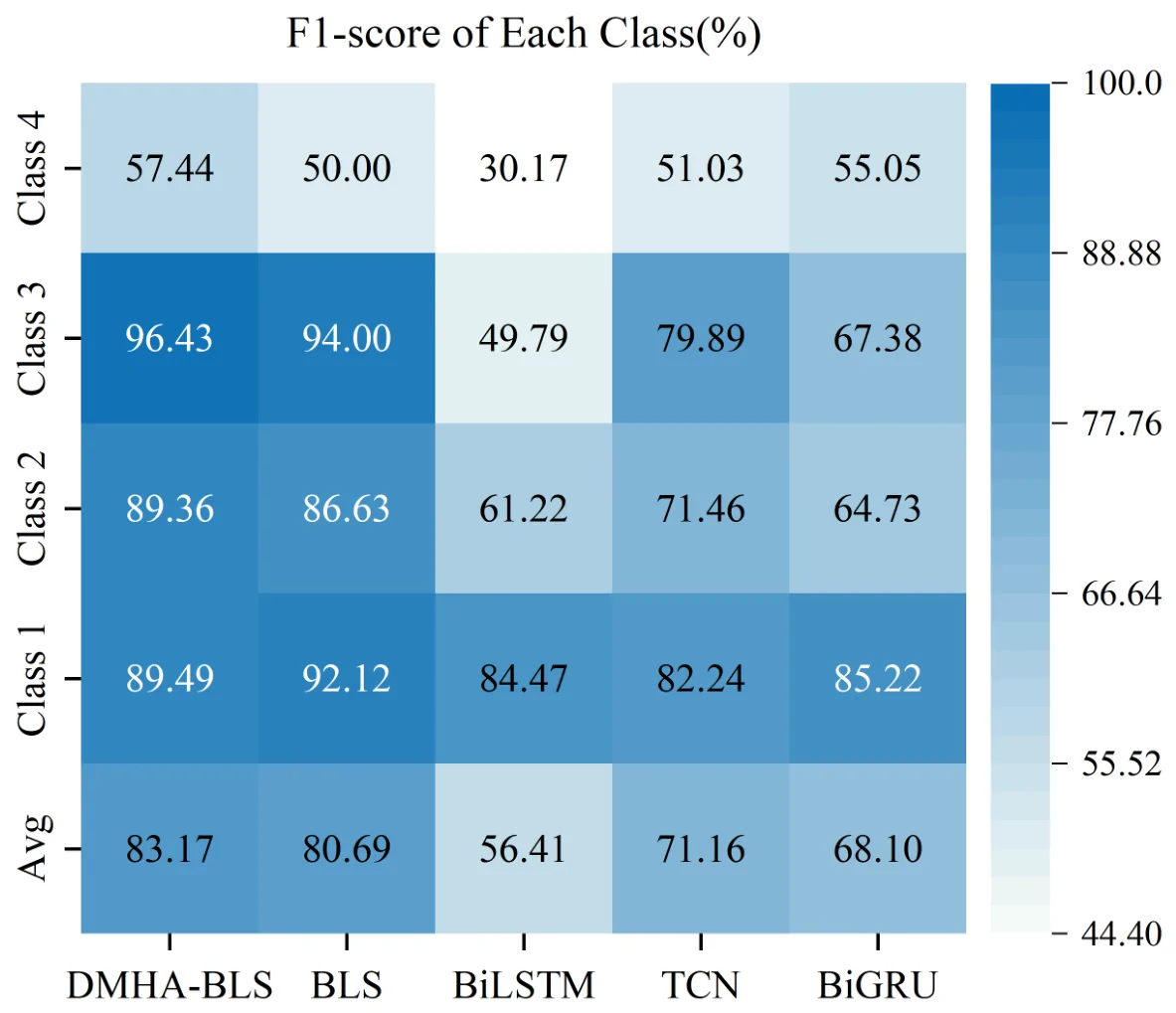
Deinterleaving performance of each class in the four-emitter scenario (200 samples).

For the primary four-emitter scenario, Table 6 reports the mean macro F1-score and standard deviation obtained over 10 independent runs at the two endpoints of the sample-size experiment. With 200 pulse samples, DMHA-BLS achieves an F1-score of 83.17±0.26%, outperforming standard BLS by 2.48 percentage points and the three temporal deep learning baselines by 12.01–26.76 percentage points. These results demonstrate the advantage of DMHA-BLS when only a limited number of labeled pulses are available. The corresponding class-wise F1-scores are shown in Figure 5.

With 8000 samples, all supervised methods achieve improved sorting performance. DMHA-BLS obtains the highest mean F1-score of 91.88±0.18%, exceeding BLS, BiLSTM, TCN, and BiGRU by 3.16, 1.23, 0.45, and 0.86 percentage points, respectively. The deterministic KNN baseline reaches 73.21% and CDIF reaches 51.71% at this sample size. Its two-sided 95% Student’s *t* confidence interval for the mean is 91.75–92.01%. Although the performance gap between DMHA-BLS and the best deep learning baseline, TCN, narrows as the sample size increases, DMHA-BLS retains the highest mean F1-score.

**Figure 6:**
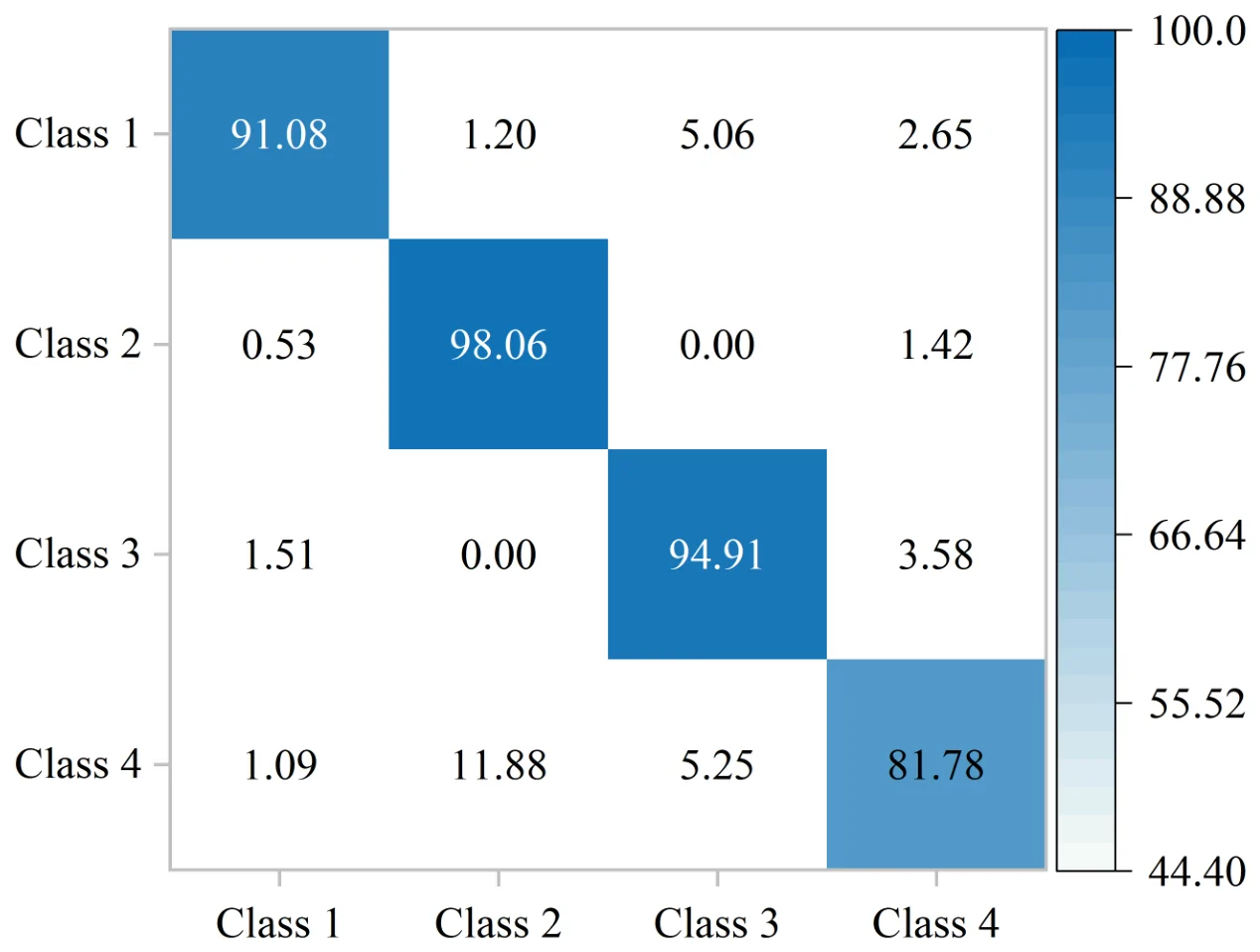
Row-normalized confusion matrix (%) of DMHA-BLS in the four-emitter scenario with 8000 pulse samples. Classes 1–4 correspond to Fixed, Jittered, Staggered, and Dwell & Switch PRI modulation, respectively.

Figure 6 presents the row-normalized confusion matrix for the 8000-sample condition. Classes 1–4 correspond to Fixed, Jittered, Staggered, and Dwell & Switch PRI modulation, respectively. DMHA-BLS identifies the Fixed, Jittered, and Staggered classes with diagonal recalls of 91.08%, 98.06%, and 94.91%, respectively. Dwell & Switch is more difficult, with an 81.78% diagonal recall; its main ambiguity is confusion with the Jittered class (11.88%), followed by the Staggered class (5.25%). This pattern is consistent with the operating scope of the method: the within-pulse PDW interactions provide strong discrimination when the four attributes jointly separate emitters, while dwell transitions can produce PDW combinations that overlap with neighboring jittered or staggered regimes. Thus, DMHA-BLS is applicable across the four modulation types, but its residual ambiguity is concentrated in the transition-rich Dwell & Switch class rather than uniformly distributed across emitters.

Figure 5 further compares the class-wise F1-scores in the 200-sample condition. DMHA-BLS achieves F1-scores of 89.49%, 89.36%, 96.43%, and 57.44% for Classes 1–4, respectively, obtaining the best performance among all methods for Classes 2, 3, and 4. The pronounced improvement achieved by DMHA-BLS for Class 4, the most difficult class for all evaluated methods, contributes substantially to the overall macro-F1 gain. In contrast, the temporal deep learning baselines exhibit greater class-wise imbalance under limited training data. BiLSTM achieves 84.47%, 61.22%, 49.79%, and 30.17% for Classes 1–4, respectively; TCN achieves 82.24%, 71.46%, 79.89%, and 51.03%; and BiGRU achieves 85.22%, 64.73%, 67.38%, and 55.05%. These results indicate that the advantage of DMHA-BLS is not limited to an easily separable emitter; instead, the proposed method provides a more balanced representation across emitters and improves the discrimination of difficult pulse classes in the small-sample regime.

**Figure 7:**
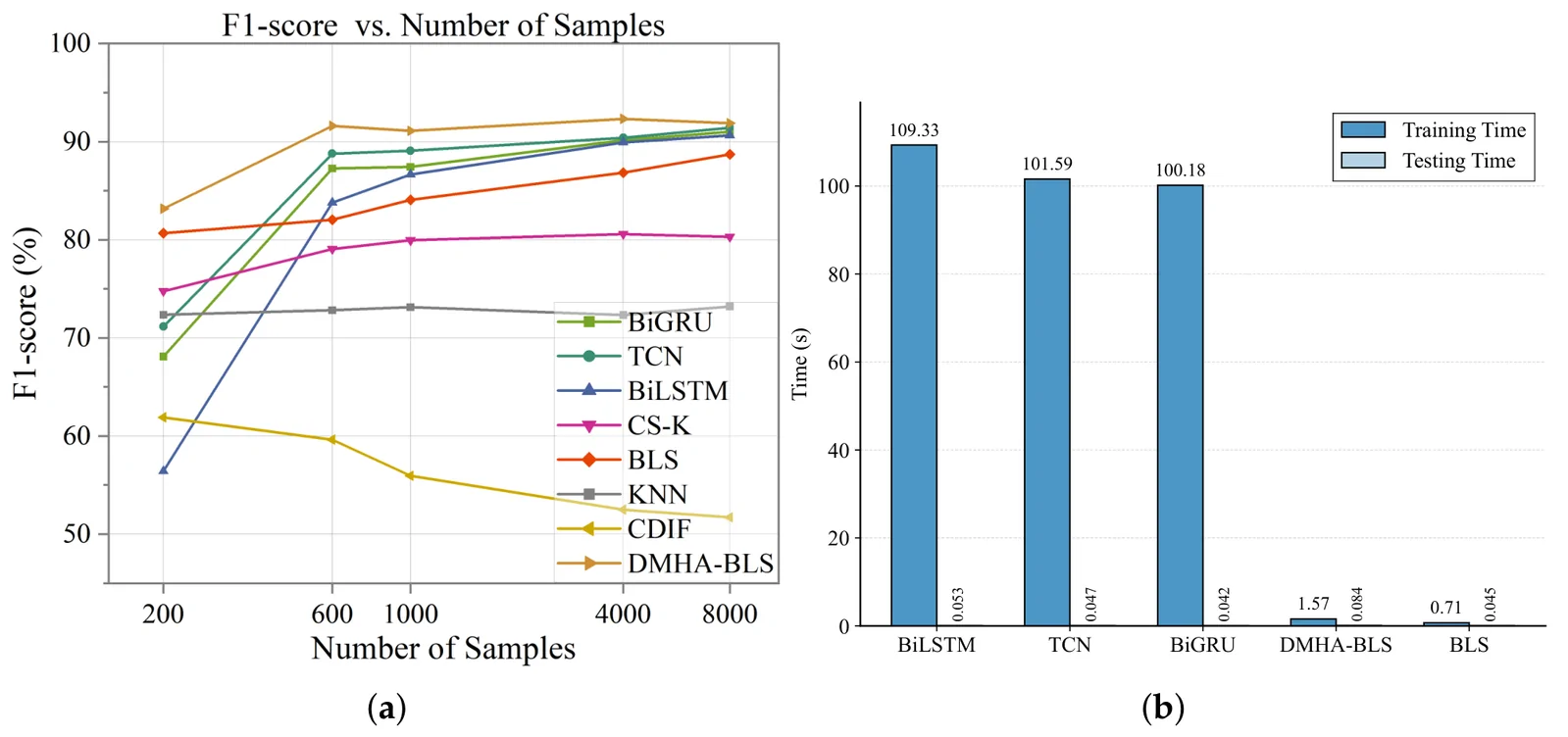
Four-emitter small-sample performance and computational efficiency. (**a**) Macro F1-score versus the number of pulse samples (logarithmic horizontal axis). (**b**) Training and testing time with 8000 samples.

Figure 7a presents the complete performance trend as the total number of pulse samples increases, including the KNN and CDIF baselines. CDIF decreases from 61.90% at 200 samples to 51.71% at 8000 samples, whereas KNN changes from 72.36% to 73.21%. Neither non-parametric/classical baseline approaches the learning-based methods in this experiment. DMHA-BLS provides its largest advantage in the most data-limited regime, whereas the gap gradually narrows as the deep learning models receive more training data. This behavior is consistent with the proposed architecture: most feature transformations are fixed rather than being estimated from labeled samples, while the orthogonality constraint preserves the input-space spectrum and the independently initialized heads provide diverse global features.

The standard deviations further show that DMHA-BLS is less sensitive to random initialization than standard BLS at both sample sizes. Therefore, DMHA-BLS provides a favorable balance between mean sorting accuracy and experimental stability, particularly when labeled pulse samples are scarce.

#### Eight-Emitter Overlapping-Parameter Scenario

To evaluate generalization beyond the primary four-emitter setting, we constructed a second, more difficult stream by chronologically interleaving 1000 pulses from eight simulated radar emitters. As summarized in Table 7, the emitters have deliberately overlapping PRI, DOA, PW, and RF ranges; seven use jittered PRI modulation, while the RF modulation is either jittered or sliding. The same chronological 4:1 split and leakage-control procedure described in Section 3.1 were used at every sample size. All model configurations were retained, except that the classifier output dimension and the CS-K cluster count were set to eight.

**Figure 8:**
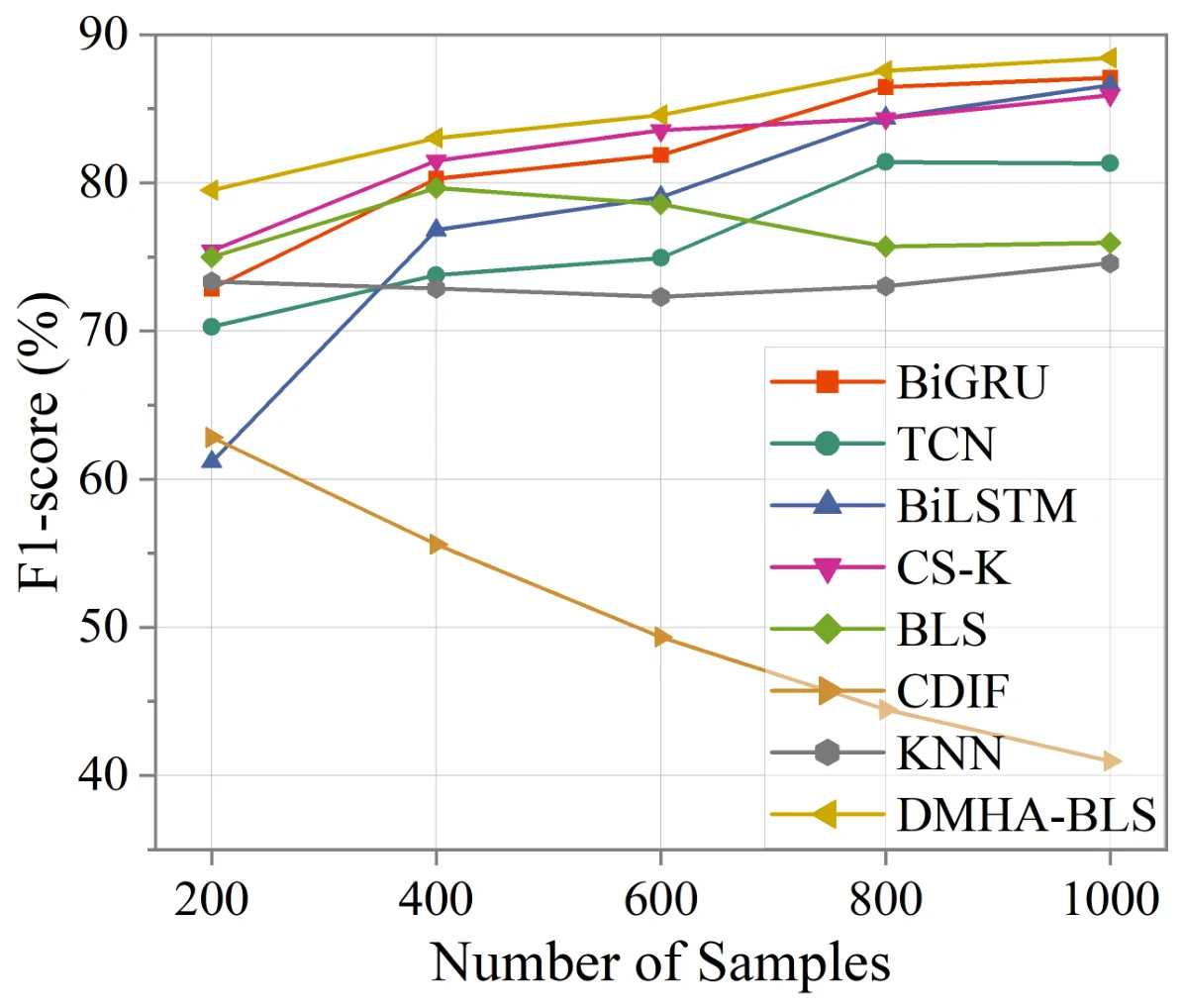
Macro F1-score versus the number of pulse samples in the eight-emitter overlapping-parameter scenario.

Figure 8 shows that DMHA-BLS achieves the highest macro F1-score at every evaluated sample size from 200 to 1000. The CDIF and KNN curves remain below the supervised learning methods throughout this overlapping-parameter experiment. BiGRU and BiLSTM approach the proposed method as more labeled pulses become available, whereas standard BLS declines after 400 samples in this setting, indicating that its shallow random mapping is less effective for the strongly overlapping classes. These results indicate that the advantage of DMHA-BLS is not restricted to the more separable four-emitter configuration, although validation on measured operational data remains necessary.

### 3.4. Training and Testing Time

Figure 7b compares runtime on the full dataset. BiLSTM, TCN, and BiGRU require 109.33, 101.59, and 100.18 s for training, respectively, whereas DMHA-BLS completes training in 1.57 s because it does not require epoch-wise backpropagation. Standard BLS is faster, requiring 0.71 s for training and 0.045 s for testing, compared with 1.57 s and 0.084 s for DMHA-BLS, respectively. Thus, relative to BLS, DMHA-BLS increases the training time by 0.86 s (2.21×) and the test-set inference time by 0.039 s (1.87×). In return, it improves the macro F1-score by 3.16 percentage points on the 8000-sample dataset, from 88.72% to 91.88%. DMHA-BLS retains a 63.8–69.6× training-time advantage over the temporal deep networks. Their batch testing times are 0.042–0.053 s, however, compared with 0.084 s for DMHA-BLS; therefore, the principal computational advantage of DMHA-BLS lies in rapid fitting and adaptation rather than the lowest batch inference time. These results indicate that DMHA-BLS offers a favorable accuracy–adaptation trade-off, whereas standard BLS or the evaluated temporal networks may remain preferable when minimum inference latency is the primary requirement.

### 3.5. Incremental Learning

For the incremental experiment, pulses were ordered chronologically, and DTOA continuity was retained across batch boundaries. The first 1000 pulses formed the initial set $D_{0}$, the next 6000 arrived in three batches of 2000, and the final 1000 were reserved as a fixed test set. Table 8 reports the results. After the third update, incremental DMHA-BLS (I-DMHA-BLS) reaches 91.78%, which is 0.06 percentage points below offline DMHA-BLS trained using the same total of 7000 pulses. Each update requires approximately 0.45 s, and the total incremental time is 1.6104 s, compared with 1.6287 s for full offline fitting. These results show that accumulated sufficient statistics can incorporate new labeled batches without replaying historical samples.

### 3.6. Robustness Under Non-Ideal Interception Conditions

Three non-ideal interception conditions were considered. The pulse-loss ratio (PLR) and spurious-pulse ratio (SPR) were independently varied from 5% to 25% in increments of 5 percentage points. In addition, a synthetic measurement-error experiment varied the measurement-error ratio from 0% to 25%. At each level, the same controlled random PDW perturbation protocol was applied before training-only normalization to every method.

**Figure 9:**
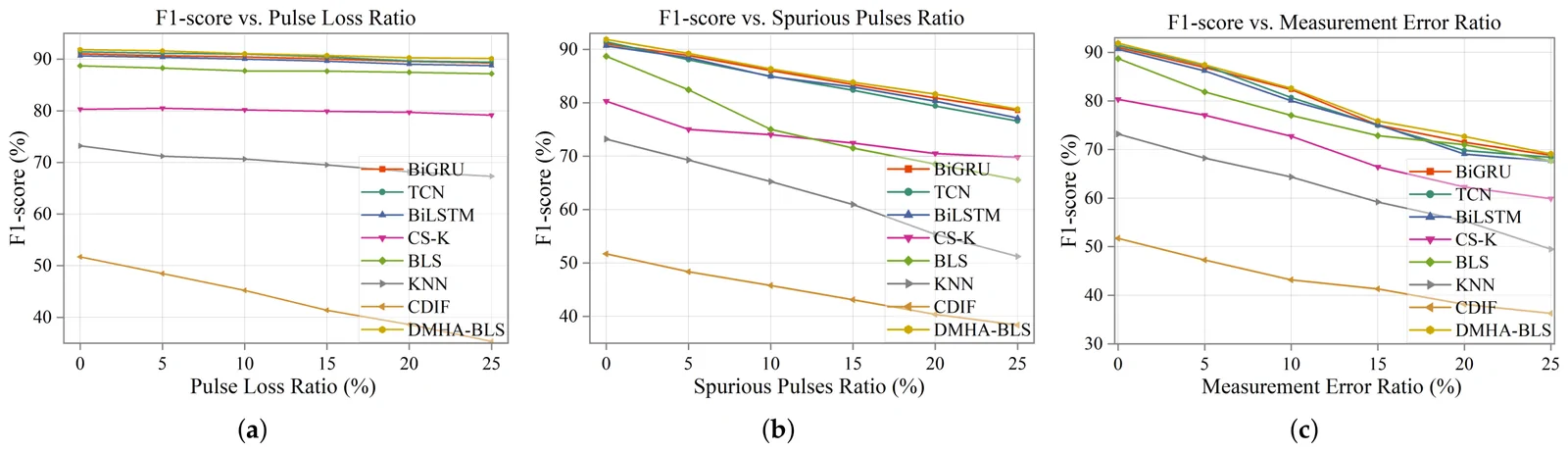
Macro F1-score under non-ideal conditions using 8000 pulse samples. (**a**) Increasing pulse-loss ratio. (**b**) Increasing spurious-pulse ratio. (**c**) Increasing PDW-level measurement-error ratio.

Figure 9a shows that DMHA-BLS maintains the highest macro F1-score throughout the evaluated PLR range. Pulse loss reduces the number of available observations and may modify DTOA-derived inputs. By jointly representing the relationships among DTOA, RF, PW, and DOA, the attention mapping retains more discriminative information than representations based primarily on individual feature directions. The residual branch further preserves the original normalized PDW information, enabling the classifier to use both the physical attributes and their joint nonlinear combinations.

Figure 9b shows a stronger overall degradation as SPR increases. BLS and TCN decline sharply, while DMHA-BLS maintains the highest reported curve through an SPR of 25%. Spurious pulses introduce additional PDWs whose parameter combinations may partially overlap those of the known emitters. The multiple independently initialized attention heads provide complementary joint representations of each pulse, reducing dependence on any single feature direction. In the simulated conditions considered here, increasing the SPR produces a larger performance degradation than increasing the PLR, while the proposed method remains the most robust under both conditions.

Figure 9c reports the PDW-level measurement-error experiment. All methods degrade as the error ratio increases, but DMHA-BLS remains the strongest curve over the tested range. KNN and CDIF are more sensitive to perturbations because KNN relies on local distances in the raw normalized space, whereas CDIF relies on timing regularities that are disrupted by perturbed inter-arrival measurements.

### 3.7. Parameter Sensitivity Analysis

**Figure 10:**
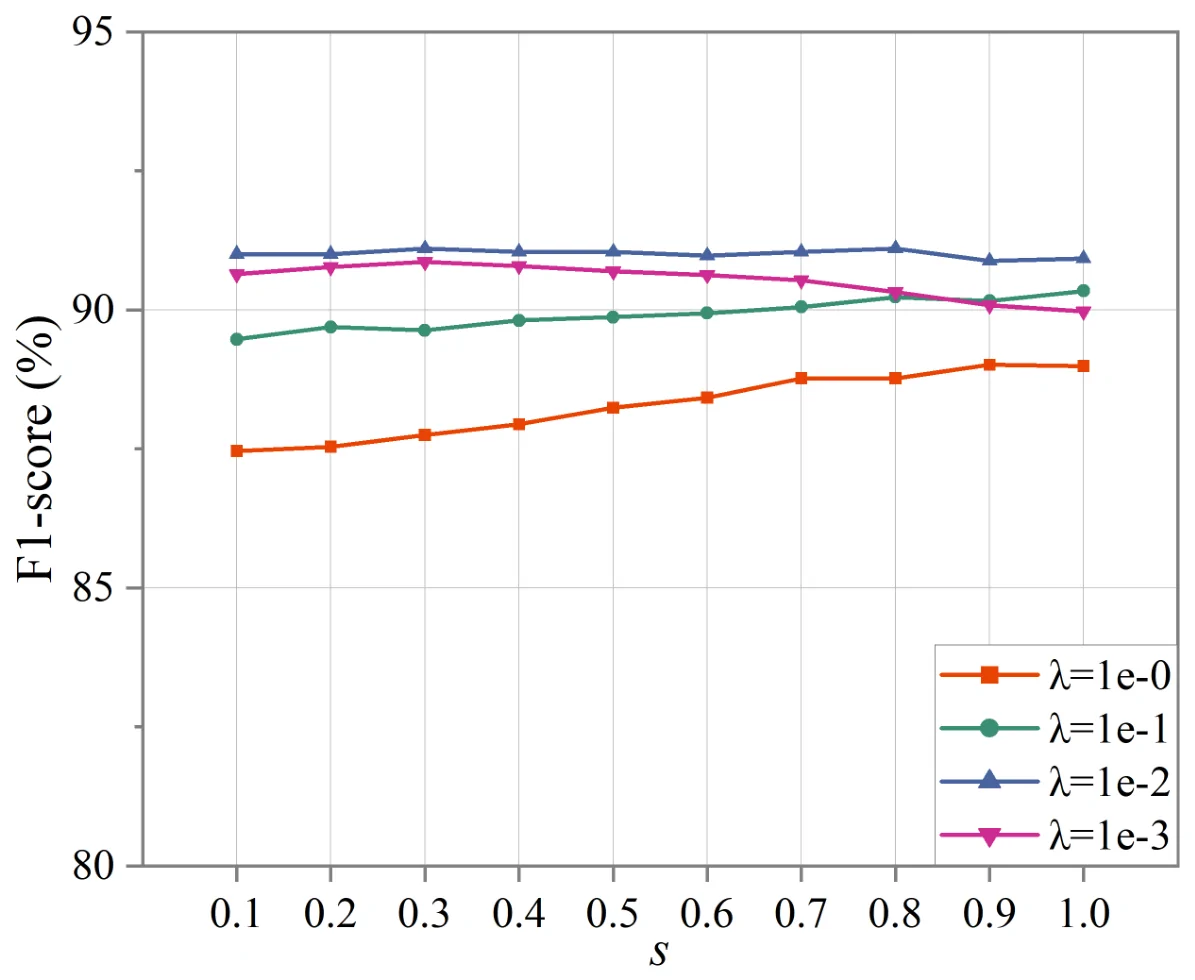
Sensitivity of the macro F1-score to the output regularization coefficient λ and enhancement scaling factor *s* using 1000 pulse samples.

The influence of the two main continuous hyperparameters was evaluated using 1000 pulse samples: the enhancement-layer scaling factor *s* in Equation (20) and the output-layer regularization coefficient λ in Equation (23). The broad feature width was fixed at 12×24=288 nodes. The factor *s* was varied from 0.1 to 1.0, while λ was evaluated over the values shown in Figure 10. In all reported experiments, s=0.8, $\lambda =10^{-2}$, and $\lambda_{\mathrm{res}}=10^{-2}$ were fixed in advance as common regularized-learning settings and were not retuned for individual sample sizes or test conditions. The residual coefficient was used for numerical stabilization of the dimension-alignment solve, not selected from the test-set sweep. Figure 10 is therefore a diagnostic sensitivity analysis rather than a parameter-selection procedure. Because the scale of $A^{⊤}A$ varies with the training-sample size, the 1000-sample result is not interpreted as identifying a universally optimal λ for every sample size.

Figure 10 shows that λ has the dominant effect on sorting performance and produces a distinct locally optimal region. Starting from a large regularization value, the macro F1-score is low; reducing λ substantially improves the score until the optimum region is reached, after which a further decrease causes a modest deterioration. This behavior follows the ridge-regression solution in Equation (23). An excessively large λ shrinks $W^{\mathrm{out}}$ toward zero and causes underfitting. Conversely, as λ approaches zero, the solution approaches unregularized least squares, becomes more sensitive to the sampled broad features, and can overfit the limited training data.

In contrast, the F1-score changes only slightly as *s* increases from 0.1 to 1.0, and the curves associated with different λ values remain comparatively flat along the *s* dimension. Within this tested interval, *s* mainly controls the scale presented to the tanh enhancement function and limits the amplitude of the enhancement nodes without materially changing their discriminatory content. DMHA-BLS is therefore relatively insensitive to *s* over the evaluated range, reducing the tuning burden for this parameter.

### 3.8. Ablation Study

Five variants were evaluated to separate the contributions of the proposed components: standard BLS; MHA-BLS, which uses one orthogonal MHA feature layer; DNMHA-BLS, which retains two orthogonal MHA blocks but omits the raw-input residual addition between them; DGMHA-BLS, which retains the complete dual-layer residual architecture but replaces the orthogonal projections with Gaussian projections; and the complete DMHA-BLS. For DNMHA-BLS, the dimension-alignment mapping is retained and the input to the second block is $F_{\mathrm{MHA}}$ rather than $F_{\mathrm{MHA}}W_{\mathrm{res}}+X$.

**Figure 11:**
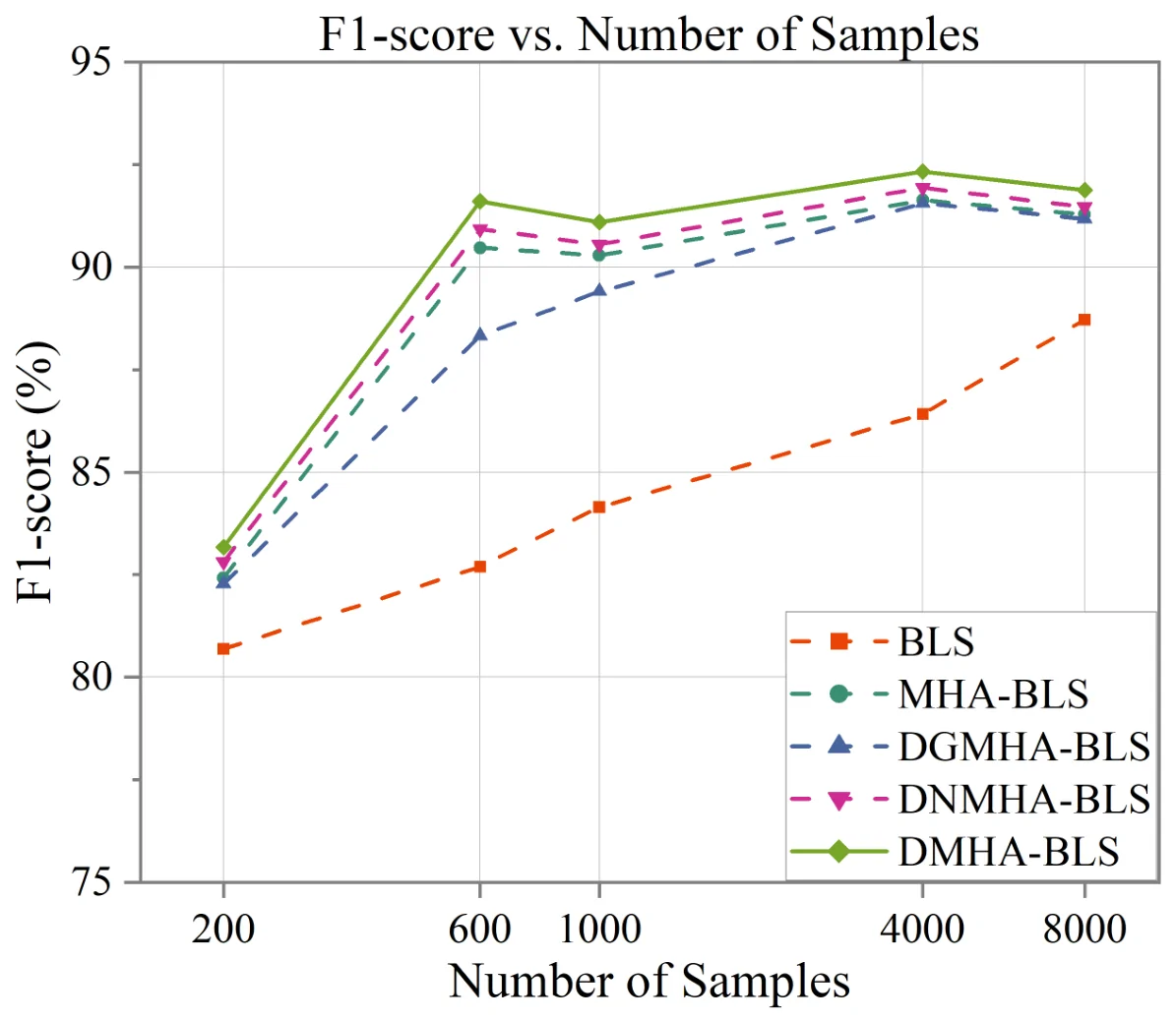
Ablation study of macro F1-score versus the number of pulse samples. MHA-BLS uses one MHA block; DNMHA-BLS uses two MHA blocks without the raw-input residual addition; DGMHA-BLS uses Gaussian projections in the complete dual-layer residual architecture; and DMHA-BLS uses orthogonal projections with the residual connection.

As shown in Figure 11, all attention-based variants outperform standard BLS. DNMHA-BLS consistently exceeds the single-layer MHA-BLS, supporting the value of the second attention block. DMHA-BLS in turn consistently exceeds DNMHA-BLS, isolating the benefit of retaining the raw PDW coordinates through the residual addition. Finally, DMHA-BLS outperforms DGMHA-BLS at every tested sample size under otherwise matched dual-layer residual architectures, which is consistent with the spectral-preservation argument in Section 2.3. These comparisons separately support the second MHA block, the inter-layer residual connection, and orthogonal initialization without attributing the entire gain to any one component. As a numerical check, the singular values of all orthogonal Q/K/V matrices remain one, whereas Gaussian projections exhibit realization-dependent singular-value spreads. Because the token matrix contains only four attribute rows, the full ${\left(SW\right)}^{⊤}\left(SW\right)$ matrix is rank-deficient and its raw condition number is dominated by numerical zero singular values. We therefore compare the effective condition number computed from the nonzero spectrum: the mean values are 9.33 for orthogonal projections and 20.99 for Gaussian projections. This supports the projection-level stability rationale.

## 4. Discussion

### 4.1. Main Findings and Accuracy–Efficiency Trade-Off

The experiments suggest that DMHA-BLS occupies a useful middle ground between conventional BLS and iteratively trained neural models. Relative to BLS, its attention features describe interactions among DTOA, RF, PW, and DOA within each pulse, which can be useful when emitter discrimination depends on joint combinations of PDW attributes rather than on individual feature dimensions. The BiLSTM, BiGRU, and TCN baselines use 16-pulse trailing windows and therefore receive temporal context in addition to the current PDW. DMHA-BLS exceeds the temporal baselines by 12.01–26.76 percentage points at 200 samples and by 0.45 percentage points over TCN at 8000 samples. The additional eight-emitter experiment further shows that the ranking is maintained when the number of emitters doubles and their PRI, DOA, PW, and RF ranges overlap substantially.

The results also clarify the cost of this design. DMHA-BLS is not uniformly faster than every shallow method: standard BLS trains and tests more quickly because it does not construct pairwise attention matrices. The practical advantage is instead rapid fitting relative to the tested deep networks while improving macro F1 over BLS.

### 4.2. Interpretation of Parameter Sensitivity

The sensitivity experiment distinguishes the roles of the two continuous hyperparameters. The strong dependence on λ is expected because this coefficient directly controls the bias–variance trade-off of the analytical output solution. Excessive regularization suppresses class-discriminative output weights, whereas insufficient regularization exposes the solution to noise and random-feature variability. By comparison, the limited change observed over s∈[0.1,1.0] indicates that the nonlinear enhancement layer maintains a similar representation over a comparatively broad scale interval. This result also suggests that the reported performance is not tied to a narrowly tuned value of *s*, which is useful when the model must be configured rapidly. However, this conclusion is restricted to the tested 1000-sample dataset and broad width of 288; sensitivity may change with the sample size, feature width, corruption level, or emitter configuration.

### 4.3. Interpretation of Robustness and Ablation Results

Modeling PDW parameters via within-pulse self-attention yields joint high-order representations that capture parameter dependencies beyond shallow mappings. The residual branch preserves original coordinates, providing complementary information.

Under pulse loss, retained PDWs still exhibit emitter-specific relationships among RF, PW, DOA, and DTOA, which the attention mechanism exploits to maintain discriminative patterns. Spurious pulses, however, cause more severe degradation because their parameter combinations can mimic known emitters or distort DTOA features, increasing representation overlap. The PDW-level measurement-error experiment shows the same overall advantage, although it does not replace receiver-level noise and calibration validation. Explicit false-pulse suppression or unknown rejection could mitigate these effects.

Ablation results confirm the value of joint modeling: MHA-BLS outperforms the shallow baseline, showing that intra-PDW interactions carry useful information. The improvement from MHA-BLS to DNMHA-BLS supports the second attention block, while the further improvement from DNMHA-BLS to DMHA-BLS supports the raw-input residual connection. On the 1600-pulse test subset, the RMS means of the normalized input branch X and the reconstructed branch $Z_{\mathrm{res}}$ were [0.3349,0.5983,0.5273,0.5998] and [0.3346,0.5981,0.5271,0.5996] for the four implemented PDW coordinates. The near equality of the first two vectors shows that the ridge-reconstructed branch is numerically compatible with the normalized raw PDW coordinate space before residual fusion. A numerical check of the fixed projections also shows that the singular values of the orthogonal Q/K/V matrices remain one, whereas Gaussian projections have realization-dependent singular values. DMHA-BLS also improves over the Gaussian-projection DGMHA-BLS, in agreement with the exact spectral-preservation property of the orthogonal projections. The complete model performs best, indicating that successive attribute-interaction extraction and preservation of the original PDW coordinates provide complementary benefits; projection conditioning is a contributing factor rather than the sole explanation.

### 4.4. Limitations and Future Work

Several limitations should be considered. First, both evaluated environments are simulated. Although the eight-emitter scenario increases class count and PDW overlap, and the measurement-error experiment applies controlled perturbations at the PDW level, the simulated distributions, modulation types, receiver errors, calibration drift, multipath, and pulse-overlap processes may not capture the diversity of measured operational data. Suitable public pulse-level datasets with emitter labels and acquisition order remain scarce, and validation on measured data with previously unseen modulation regimes is required before deployment claims can be made. Second, its computational cost nevertheless increases with the number of heads, the embedded feature dimension, and the broad width. Evaluation under substantially higher pulse arrival rates and hardware-optimized vectorized implementations is therefore still required. Third, the current supervised formulation assumes a fixed known emitter label set and does not address open-set discovery, emitter appearance or disappearance, or concept drift.

These limitations motivate three immediate extensions: evaluation on measured multi-receiver data; vectorized and hardware-optimized self-attention for higher pulse arrival rates; and open-world incremental sorting that detects novel emitters while adapting to non-stationary PDW statistics.

## 5. Conclusions

This article proposes DMHA-BLS, a rapid framework for radar signal sorting under limited labeled data and latency-sensitive operating conditions. The method replaces conventional BLS mappings with two fixed random-orthogonal attention blocks, preserves the original PDW attributes through a closed-form residual projection, and estimates the classifier analytically. On the simulated four-emitter stream, DMHA-BLS attains 83.17% macro F1 with 200 samples and 91.88% with 8000 samples. It also remains top-performing from 200 to 1000 samples in the eight-emitter scenario with strongly overlapping PDW ranges. Its full-data training time is 1.57 s, compared with 100–109 s for the evaluated deep networks using 16-pulse trailing windows. These results indicate a practical balance between sorting performance, small-sample behavior, and analytical adaptation efficiency. Future work will focus on measured data, scalable attention, false-pulse rejection, and open-set adaptation in non-stationary emitter environments.

## Figures and Tables

**Table 1:** Parameters of the simulated radar emitters.

Radar	PRI (μs)	PW (μs)	RF (GHz)	DOA (°)
1	520 fixed	10–30	8.10–8.40 jittered	34–37
2	350, J=0.25 jittered	30–50	8.15–8.30 jittered	36–39
3	100, 280, 800 staggered	20–40	8.35–8.60 jittered	33–37
4	400×15,600×20 900×10 dwell and switch	25–45	8.25–8.50 jittered	36.5–38

**Table 2:** The core parameters of DMHA-BLS and standard BLS.

Method	Parameters	Value
DMHA-BLS	Feature/enhancement heads, h/m	12/12
	Per-head dimension, $d_{k}$	12
	Concatenated broad width, *p*	288
	Residual regularization, $\lambda_{\mathrm{res}}$	0.01
	Bias distribution, β	U(−1,1)
	scaling factor, *s*	0.8
	Output regularization, λ	0.01
BLS	Feature windows	24
	Feature nodes per window	36
	Enhancement nodes	800
	Shrinkage factor	0.8
	Output regularization	0.01

**Table 3:** The core parameters of the deep learning baselines.

Parameters	TCN	BiLSTM	BiGRU
Input tensor shape	(B,4,16)	(B,16,4)	(B,16,4)
Core architecture	Two temporal blocks	Two-layer BiLSTM	Two-layer BiGRU
Channel/hidden width	[32,64]	64 per direction	64 per direction
Kernel size	3	–	–
Dilation factors	[1,2]	–	–
Activation	ReLU	LSTM gates	GRU gates
Dropout rate	0.3	0.3	0.3
Feature aggregation	Final-position output	Final-position output	Final-position output
Classification layer	64→C	128→C	128→C
Loss function	Cross-entropy	Cross-entropy	Cross-entropy
Optimizer	Adam	Adam	Adam
Initial learning rate	$10^{-4}$	$10^{-4}$	$10^{-4}$
Batch size	32	32	32
Training epochs	200	200	200

**Table 4:** Architecture-level parameter and complexity comparison at C=4. *N* is the number of training pulses, E=200 is the number of deep learning epochs, and $p_{\mathrm{BLS}}=1664$ is the standard-BLS broad width.

Method	Fixed/Trainable or Solved Scalars	Total FP32 Storage	Training Cost	Per-Pulse Inference Cost
DMHA-BLS	10,608 fixed/1728 solved	12,336 (≈48 KiB)	$O(Nq^{2}+q^{3}+Np^{2}+p^{3})$	$\approx 5.1\times 10^{4}+pC$ multiplies
BLS	696,320 fixed/6656 solved	702,976 (≈2.68 MiB)	$O(Np_{\mathrm{BLS}}^{2}+p_{\mathrm{BLS}}^{3})$	feature mapping + enhancement + $p_{\mathrm{BLS}}C$
TCN	0/24,612 trainable	24,612 (≈96 KiB)	$O(ENLP_{\mathrm{TCN}})$	$O(LP_{\mathrm{TCN}})$
BiLSTM	0/135,684 trainable	135,684 (≈530 KiB)	$O(ENLP_{\mathrm{LSTM}})$	$O(LP_{\mathrm{LSTM}})$
BiGRU	0/101,892 trainable	101,892 (≈398 KiB)	$O(ENLP_{\mathrm{GRU}})$	$O(LP_{\mathrm{GRU}})$

**Table 5:** The core parameters of the CS-K clustering baseline.

Parameters	Value
Distance metric	Euclidean distance
Number of clusters	*C* (number of emitters)
Labeled seed ratio	10% per class
Initial cluster centers	Class-wise means of labeled seeds
Maximum iterations	300
Convergence tolerance	$10^{-4}$
Cluster-label alignment	Hungarian algorithm

**Table 6:** Macro F1-score (%) in the four-emitter scenario at two sample sizes. Results are reported as the mean ± standard deviation over 10 independent runs; the final row gives two-sided 95% Student’s *t* confidence intervals for the mean at 8000 samples.

Samples	DMHA-BLS	BLS	BiLSTM	TCN	BiGRU
200	**83.17 ± 0.26**	80.69 ± 0.54	56.41 ± 7.29	71.16 ± 3.14	68.10 ± 3.36
8000	**91.88 ± 0.18**	88.72 ± 0.43	90.65 ± 0.31	91.43 ± 0.25	91.02 ± 0.27
8000 (95% CI)	**91.75–92.01**	88.41–89.03	90.43–90.87	91.25–91.61	90.83–91.21

**Table 7:** Parameters of the eight simulated radar emitters in the overlapping-parameter scenario.

Radar	PRI (μs)	PRI Modulation	DOA (°)	PW (μs)	PW Modulation	RF (GHz)	RF Modulation
1	52	Fixed	88–106	0.55–0.85	Jittered	11.75–12.15	Sliding
2	49–57	Jittered	94–112	0.08–0.18	Jittered	12.15–12.45	Jittered
3	52–54	Jittered	72–94	0.75–1.05	Jittered	12.05–12.40	Jittered
4	55–58	Jittered	78–100	0.80–1.10	Jittered	11.90–12.25	Jittered
5	44–50	Jittered	84–106	0.85–1.15	Jittered	12.20–12.55	Jittered
6	45–48	Jittered	64–88	0.50–0.90	Jittered	11.85–12.35	Sliding
7	50–56	Jittered	86–110	0.08–0.20	Jittered	11.95–12.25	Jittered
8	44–47	Jittered	68–92	0.85–1.20	Jittered	11.95–12.30	Jittered

**Table 8:** Incremental learning performance and computation time.

Algorithm	Samples	F1 (%)	Train/Update Time (s)	Total Time (s)
DMHA-BLS	1000	90.78 ± 0.24	0.2622 ± 0.0057	0.2622
DMHA-BLS	7000	91.84 ± 0.20	1.6287 ± 0.0265	1.6287
I-DMHA-BLS	1000→3000	91.16 ± 0.23	0.4489 ± 0.0091	0.7111
I-DMHA-BLS	3000→5000	91.55 ± 0.22	0.4496 ± 0.0107	1.1607
I-DMHA-BLS	5000→7000	91.78 ± 0.19	0.4497 ± 0.0116	1.6104

## Data Availability

The original data presented in this study are openly available in Figshare at https://doi.org/10.6084/m9.figshare.33327453.

## Abbreviations

The following abbreviations are used in this manuscript:

BiGRU	Bidirectional gated recurrent unit
BiLSTM	Bidirectional long short-term memory
BLS	Broad learning system
CDIF	Cumulative difference histogram
CS-K	Constrained-seed K-means
DL	Deep learning
DGMHA-BLS	Dual-layer Gaussian multi-head attention broad learning system
DMHA-BLS	Dual-layer multi-head attention broad learning system
DNMHA-BLS	Dual-layer no-residual multi-head attention broad learning system
DOA	Direction of arrival
DTOA	Difference of time of arrival
ESM	Electronic support measures
MHA	Multi-head attention
MHA-BLS	Multi-head attention broad learning system
KNN	K-nearest neighbors
PA	Pulse amplitude
PDW	Pulse description word
PLR	Pulse-loss ratio
PRI	Pulse repetition interval
PW	Pulse width
RF	Radio frequency
RSS	Radar signal sorting
SDIF	Sequential difference histogram
SPR	Spurious-pulse ratio
TCN	Temporal convolutional network
TOA	Time of arrival
